# Supplementary material for: Myeloid-derived miR-6236 potentiates adipocyte insulin signaling and prevents hyperglycemia during obesity
Source: Nat Commun. 2024 Jun 25;15:5394. doi: 10.1038/s41467-024-49632-z (PMC11199588; doi:10.1038/s41467-024-49632-z)
Supplement: Supplementary file 1 — Supplementary Information [file 41467_2024_49632_MOESM1_ESM.pdf]

Myeloid-derived miR-6236 potentiates adipocyte insulin signaling and prevents hyperglycemia during obesity.

Authors: Bam D. Paneru[1]*, Julia Chini[1,2]*, Sam J. McCright[1,2], Nicole DeMarco[1], Jessica Miller[1], Leonel D. Joannas[3], Jorge Henao-Mejia[3], Paul M. Titchenell[4], David M. Merrick[5], Hee-Woong Lim[6], Mitchell A. Lazar[5,7], David A. Hill[1,7,8]

Corresponding Author:
David A. Hill, MD, PhD
Division of Allergy and Immunology
Children's Hospital of Philadelphia
Abramson Research Building, 1208B
3615 Civic Center Blvd., Philadelphia, PA 19104
Phone: (215) 590-2549
E-mail: hilld3@chop.edu

Paneru & Chini *et al* - Supplementary Figure 1

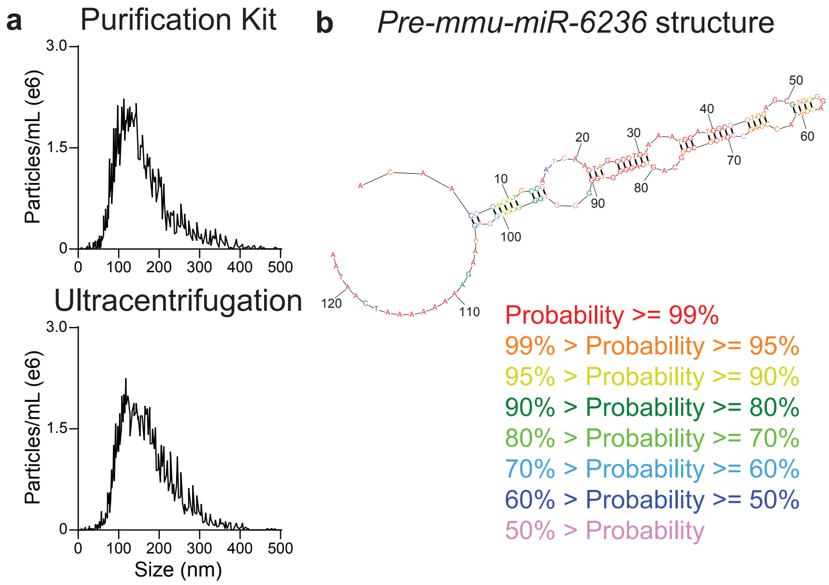

c *mmu-miR-6236* sequence isoforms

Custom ID	Mature sequence	BP	Read count	RPM
miR-6236a	CACCTGCCGAATCAACTAGC	20	70	4.07
miR-6236b	TCACCTGCCGAATCAACTAGC	21	65	3.78
miR-6236c	CGAATCAACTAGCCCTGAAA	20	65	3.78
miR-6236d	AAAATGGATGGCGCTGGAGC	20	65	3.78
miR-6236e	CCTGAAAATGGATGGCGCTGGAGC	24	57	3.32
miR-6236f	TGAAAATGGATGGCGCTGGAGC	22	48	2.79
miR-6236g	CTCACCTGCCGAATCAACTAGC	22	47	2.73
miR-6236h	GAAAATGGATGGCGCTGGAGC	21	43	2.50
miR-6236i	ACTCACCTGCCGAATCAACTAGC	23	41	2.39
miR-6236j	GCCGAATCAACTAGCCCTGA	20	41	2.39
miR-6236k	AACTCACCTGCCGAATCAACTAGC	24	38	2.21
miR-6236l	CTGAAAATGGATGGCGCTGGAGC	23	37	2.15
miR-6236m	CCCTGAAAATGGATGGCGCTG	21	35	2.04
miR-6236n	TGCCGAATCAACTAGCCCTGAAA	23	35	2.04
miR-6236o	CTGCCGAATCAACTAGCCCT	20	19	1.11
miR-6236p	ACAACTCACCTGCCGAATCAAC	22	19	1.11
miR-6236q	TAGCCCTGAAAATGGATGGCGCTGGAGC	28	16	0.93
miR-6236r	CCTGCCGAATCAACTAGCCCT	21	14	0.81

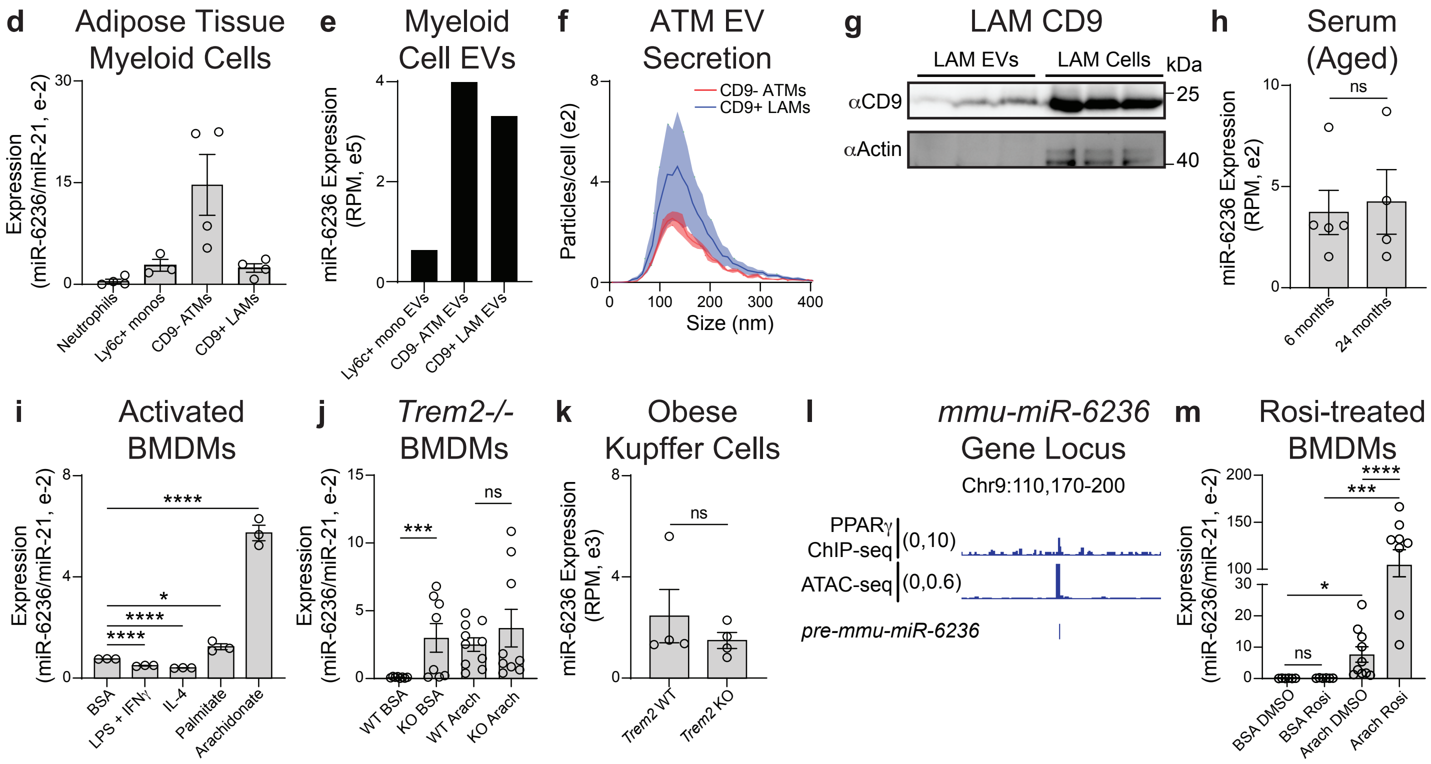

Paneru & Chini *et al* - Supplementary Figure 1

Supplementary Fig. 1: Pre-miR-6236 secondary structure, mature sequence isoforms, and expression characteristics. a, Nanoparticle Tracking Analysis (NTA) of macrophage-derived extracellular vesicles (EVs) isolated via purification kit or ultracentrifugation. b, Stem-loop structure of pre-mmu-miR-6236. c, Most abundant mature sequence isoforms in adipose tissue small RNA-seq libraries (BP = base pairs, RPM = reads per million). d, miR-6236 expression in sort-purified myeloid immune cells from eWAT from DIO male mice (Ly6c+ Monocytes, n = 3 mice, Neutrophils, CD9- ATMs, and CD9+ LAMs, n = 4). e, miR-6236 expression in eWAT macrophage and monocyte (mono) -derived EVs of DIO male mice. f, NTA of eWAT macrophage-derived EVs (n = 4 biological replicates/group). g, Non-reducing CD9 Western blot in EV and cellular fractions of LAMs from DIO male mice (n = 3 biological replicates/group). h, miR-6236 expression in serum of young (6 month; n = 5) and aged (24 month; n = 4) mice. i, miR-6236 expression in BMDMs under different activation conditions (n = 3; BSA vs. LPS + IFNγ, $p < 0.0001$; BSA vs. IL-4, $p = 0.0001$; BSA vs. Palmitate, $p = 0.014$; BSA vs. Arachidonate, $p < 0.0001$). j, miR-6236 expression in Trem2 sufficient (WT) or deficient (KO) BMDMs in the absence (BSA) or presence of arachidonate (Arach) (WT, n = 8 technical replicates; KO BSA, n = 9 technical replicates; KO Arach, n = 10 technical replicates) (WT BSA vs. KO BSA, $p = 0.0005$). k, miR-6236 expression in DIO, *Trem2* WT or KO Kupffer cells (n = 4). l, PPAR γ ChIP-seq browser tracks in macrophages and ATAC-seq tracks in CD9+ LAMs at the miR-6236 gene locus. m, miR-6236 expression in BMDMs treated with rosiglitazone (Rosi) in the absence (n = 6 technical replicates/group) or presence (n = 10 technical replicates/group) of arachidonate (BSA DMSO vs. Arach DMSO, $p = 0.033$; BSA Rosi vs. Arach Rosi, $p = 0.0002$; Arach DMSO vs. Arach Rosi, $p < 0.0001$). Data presented as mean ± SEM where appropriate; ns = not significant, *$p < 0.05$, ***$p < 0.001$, ****$p < 0.0001$, two-tailed Student's t test.

Paneru & Chini *et al* - Supplementary Figure 2

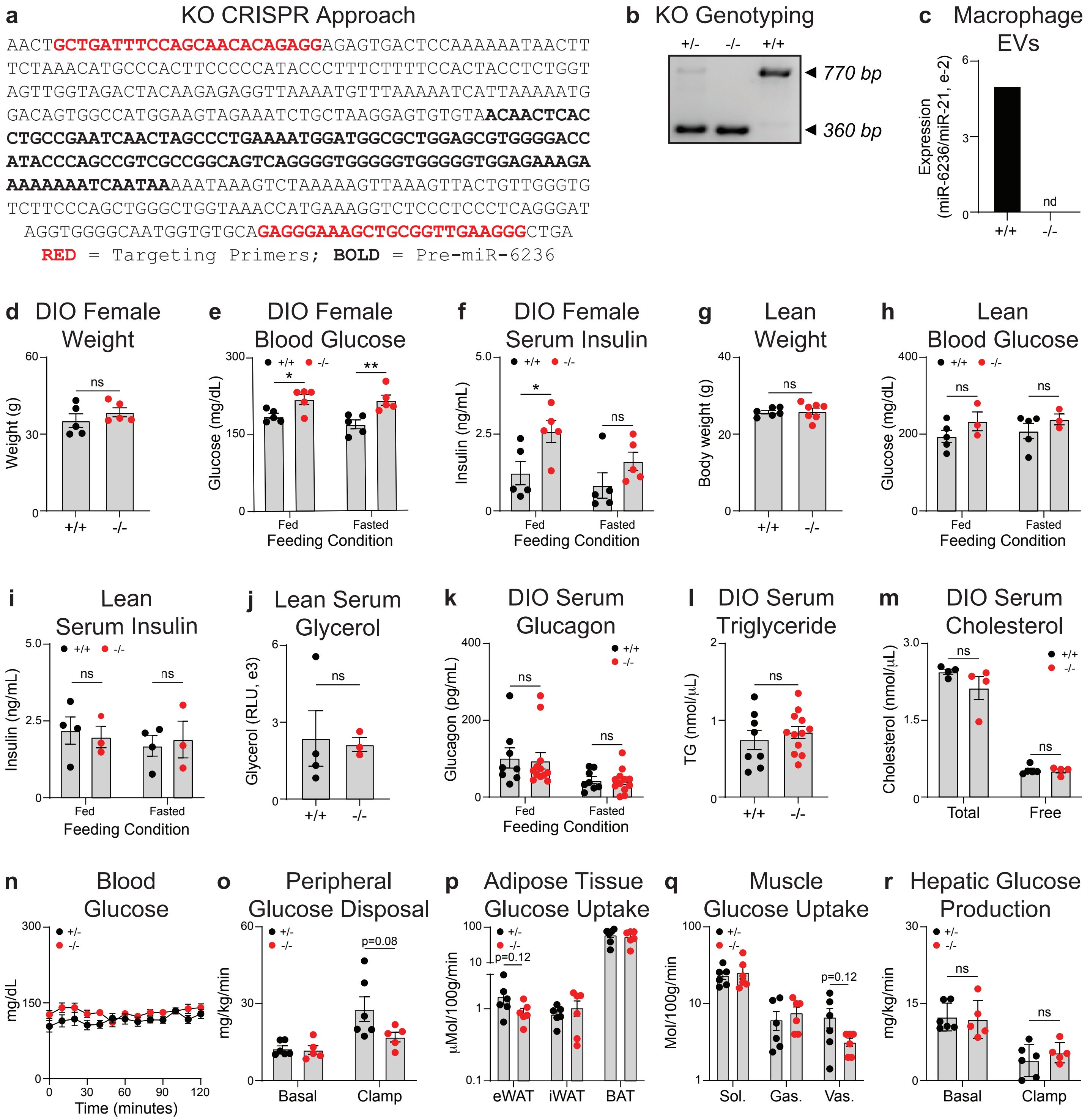

Paneru & Chini *et al* - Supplementary Figure 2

Supplementary Fig. 2: Development and metabolic phenotype of miR-6236 KO mice. a, Genomic sequence of miR-6236 showing pre-miR-6236 sequence (bold) and flanking sequences. Region between CRISPR targeting primers (red) is deleted in whole body knockout mice. b, Genotyping gel image showing expected band size of the miR-6236 DNA locus in heterozygous (+/-), knockout (-/-), and wild-type (+/+) mice. c, miR-6236 expression in EVs of BMDMs. d, Body weight of DIO female mice fed high fat diet for 12 weeks (n = 5). e-f, Fed and fasted (5 hrs.) blood glucose level (e) and serum insulin level (f) in DIO female mice (n = 5; Fed Glucose, p = 0.023; Fasted Glucose, p = 0.0080; Fed Insulin, p = 0.033). g, Body weight of lean wild-type (n = 6) and knockout (n = 7) male mice fed normal chow diet for 18 weeks. h, Fed and fasted (5 hrs.) blood glucose level in lean wild-type (n = 5) and knockout (n = 3) male mice. i, Fed and fasted serum insulin levels in lean wild-type (n = 4) and knockout (n = 3) male mice. j, Serum glycerol level in lean wild-type (n = 4) and knockout (n = 3) male mice. k-l, Serum glucagon (k) and triglyceride (l) in DIO wild-type (n = 8) and knockout (n = 12) male mice. m, Serum cholesterol in DIO male mice (n = 4). n-r, Blood glucose (n), peripheral glucose disposal (o), adipose tissue glucose uptake (p), muscle glucose uptake (q), and hepatic glucose production (r) as determined during a hyperinsulinemic-euglycemic clamp in fasted DIO male mice of +/- (n = 6) or -/- (n = 5) genotypes. Data representative of ≥2 experiments with exception of clamp which is 1 experiment. Data presented as mean ± SEM; nd = not detected, ns = not significant, *p < 0.05, **p < 0.01, two-tailed Student’s t test.

Paneru & Chini *et al* - Supplementary Figure 3

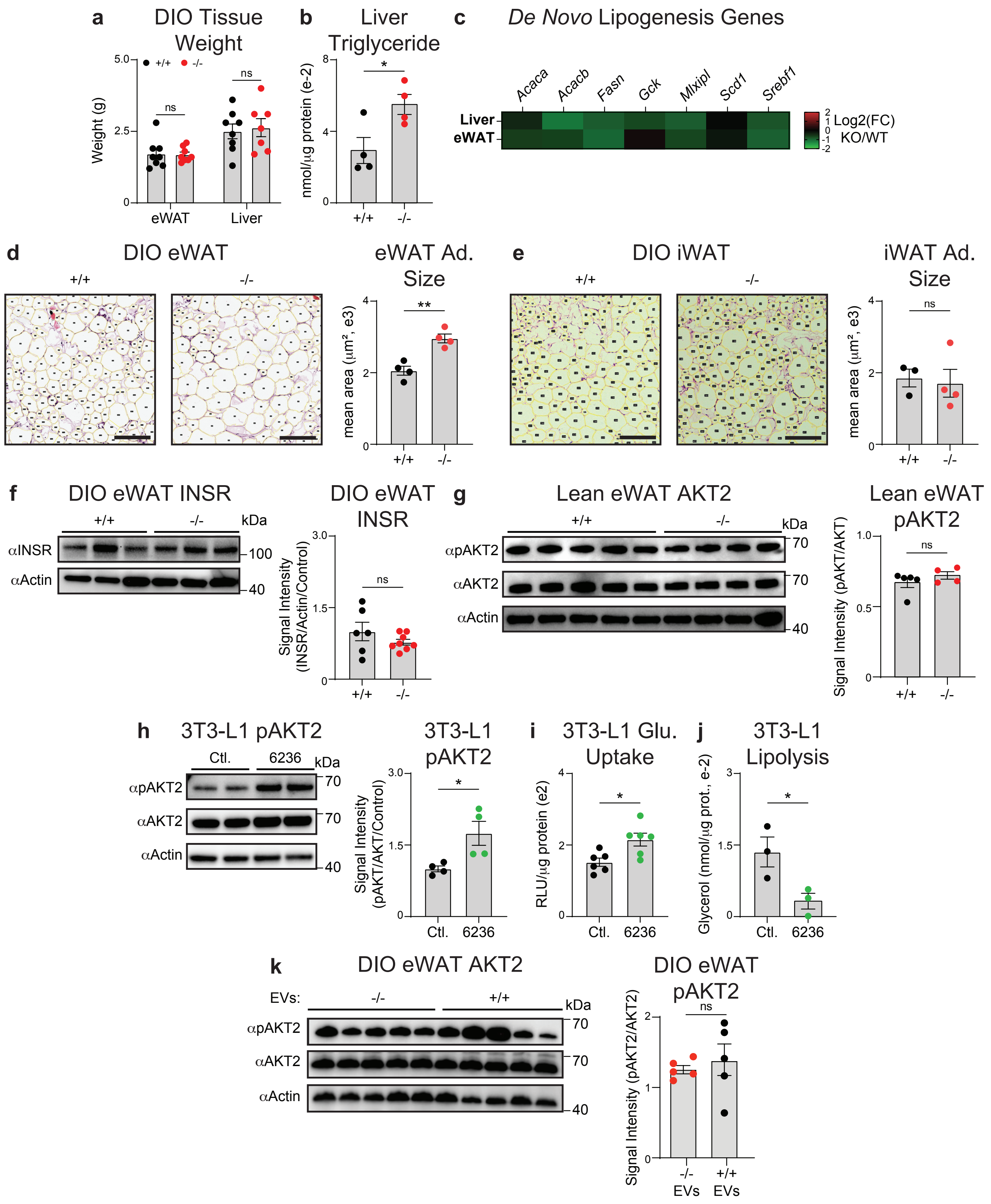

Supplementary Fig. 3: Adipose tissue and liver features in miR-6236 KO mice. a, eWAT and liver weight of DIO wild-type (+/+; n = 8) or miR-6236 knockout (-/-; n = 7) male mice. b, Triglyceride content of liver from DIO male mice (n = 4/group; p = 0.031). c, Heatmap of *de novo* lipogenesis-related gene expression in liver and eWAT of DIO +/+ or -/- male mice fed *ad libitum* (n = 4/group). d, H&E sections and adipocyte size calculations from eWAT of DIO male mice (n = 4; bar = 200 μm; p = 0.0022). e, H&E sections and adipocyte size calculations from iWAT of DIO wild-type (n = 3) and knockout (n = 4) male mice (bar = 200 μm). f, Representative insulin receptor (INSR) Western blot and quantification from two independent experiments in eWAT of DIO wild-type (n = 6) and knockout (n = 8) male mice. g, AKT2 and pAKT2 Western blot and quantification in eWAT of lean wild-type (n = 5) and knockout (n = 4) male mice. h, Representative AKT2 and pAKT2 Western blot and quantification from two independent experiments in 3T3-L1 adipocytes transfected with miR-6236 mimic or scrambled control (Ctl.) (n = 4/group; p = 0.028). i, Insulin-stimulated glucose uptake in 3T3-L1 adipocytes transfected with miR-6236 mimic or control (n = 6/group; p = 0.014). j, Glycerol release from 3T3-L1 adipocytes transfected with miR-6236 mimic or control in the presence of insulin (n = 3/group, p = 0.043). k, AKT2 and pAKT2 Western blot and quantification in DIO +/+ male mice injected i.p. with EVs from -/- or +/+ BMDMs (n = 5 mice/group). Abbreviations: H&E: Hematox-ylin & Eosin. Data representative of ≥2 experiments. Data presented as mean ± SEM; ns = not significant, *p < 0.05, **p < 0.01, two-tailed Student's t test.

Paneru & Chini *et al* - Supplementary Figure 4

a

Predicted Target	Binding Sites
Prkca	10
Pten	7
Enpp1	5
Pid1	5
Pip4k2b	5
Prkaa1	5
Ptprj	5
Gpr21	4
Grb10	4
Irs1	4

b *Pten* 3'UTR with predicted *miR-6236* binding sites in red

5′-GAAGT**TTTTCAG**TAGAAATTGTCCTACAGATGCTTTATCGACTTGCTATTGGAAGAATAGATCTTCTTAAAT
GTGCAGTGTTGAGTCACTTCGTTATAGTGGTAGAGTTGGGATTAGGGCTTCAATTTTACTTCTTAAATATCAT
TCTATGTTTGATATGCCCAGACTGCATACAATTTAAAGCAAGAGTACAACTACTATCGTAATGGTAATGTGAAGAT
GCTATTACAAAGGATCTCCTCCCAACCCCTCGGGAATTTGGTGTCTTTCAAATTATATCTTGACCTTGACATTTGAA
TATCCAGCCATTATTAGATTTCTTAATGGTGTGAAGTC**CCATTTTC**AATAA-3′

c Hypothetical *miR-6236* variant binding to *Pten* 3'UTR

miR-6236e: 3′-CGAGGTCGCGGTAGGT**AAAAGTC**C-5′
Predicted binding site 1: 5′-AATAAGGCATTGAAGT**TTTTCAG**T-3′

miR-6236f: 3′-CGAGGTCGCGGTA**GGTAAAAG**T-5′
Predicted binding site 2: 5′-ATGGTGTGAAGTC**CCATTTTC**A-3′

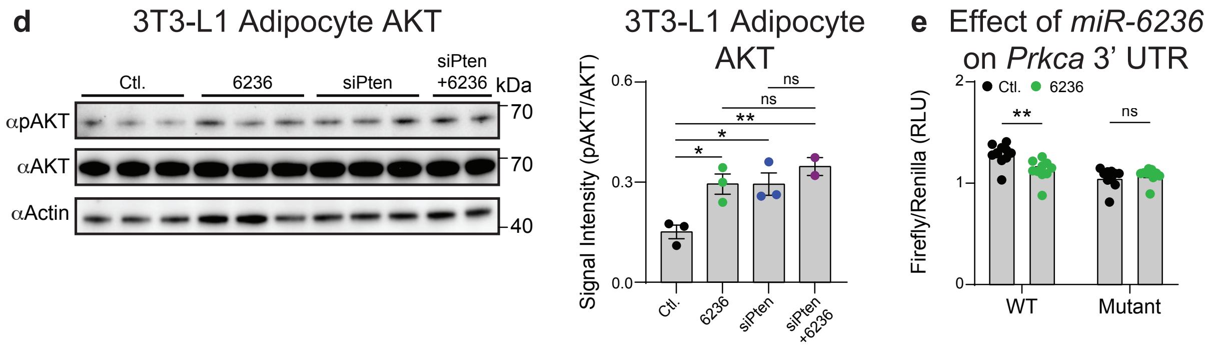

Supplementary Fig. 4: miR-6236 binding sites in *Pten* 3'UTR, regulation of AKT2 phosphorylation by miR-6236 mimic and *Pten* siRNA, and effects of miR-6236 mimic on *Prkca* 3' UTR-mediated expression. a, Predicted miR-6236 targets with binding site number. b, Portion of *Pten* 3'UTR cloned into pmirGLO for target validation. Red: predicted miR-6236 binding sites. c, Hypothetical miRNA-6236 : *Pten* 3'UTR binding. d, AKT2 and pAKT2 Western blot and quantification in 3T3-L1 transfected with scrambled control RNA (Ctl., n = 3), 6236 mimic (6236; n = 3), *Pten* siRNA (siPten; n = 3), and *Pten* siRNA + 6236 mimic (siPten + 6236; n = 2) (Ctl. vs. 6236, p = 0.017; Ctl. vs. siPten, p = 0.022; Ctl. vs. siPten +6236, p = 0.0096). e, Dual luciferase reporter assay of scrambled control RNA (Ctl.) or miR-6236 (6236) binding to wild-type (WT) or miR-6236 binding sites-deleted (Mutant) *Prkca* 3'UTR cloned in pmirGLO plasmid downstream of firefly ORF (n = 10; WT Ctl. vs. 6236, p = 0.0038). Abbreviations: Ctl: scrambled control; 6236: miR-6236 mimic; siPten: Pten siRNA. Data representative of ≥2 experiments. Data presented as mean ± SEM; ns = not significant, *p < 0.05, **p < 0.01, two-tailed Student's t test.

Paneru & Chini *et al* - Supplementary Figure 5

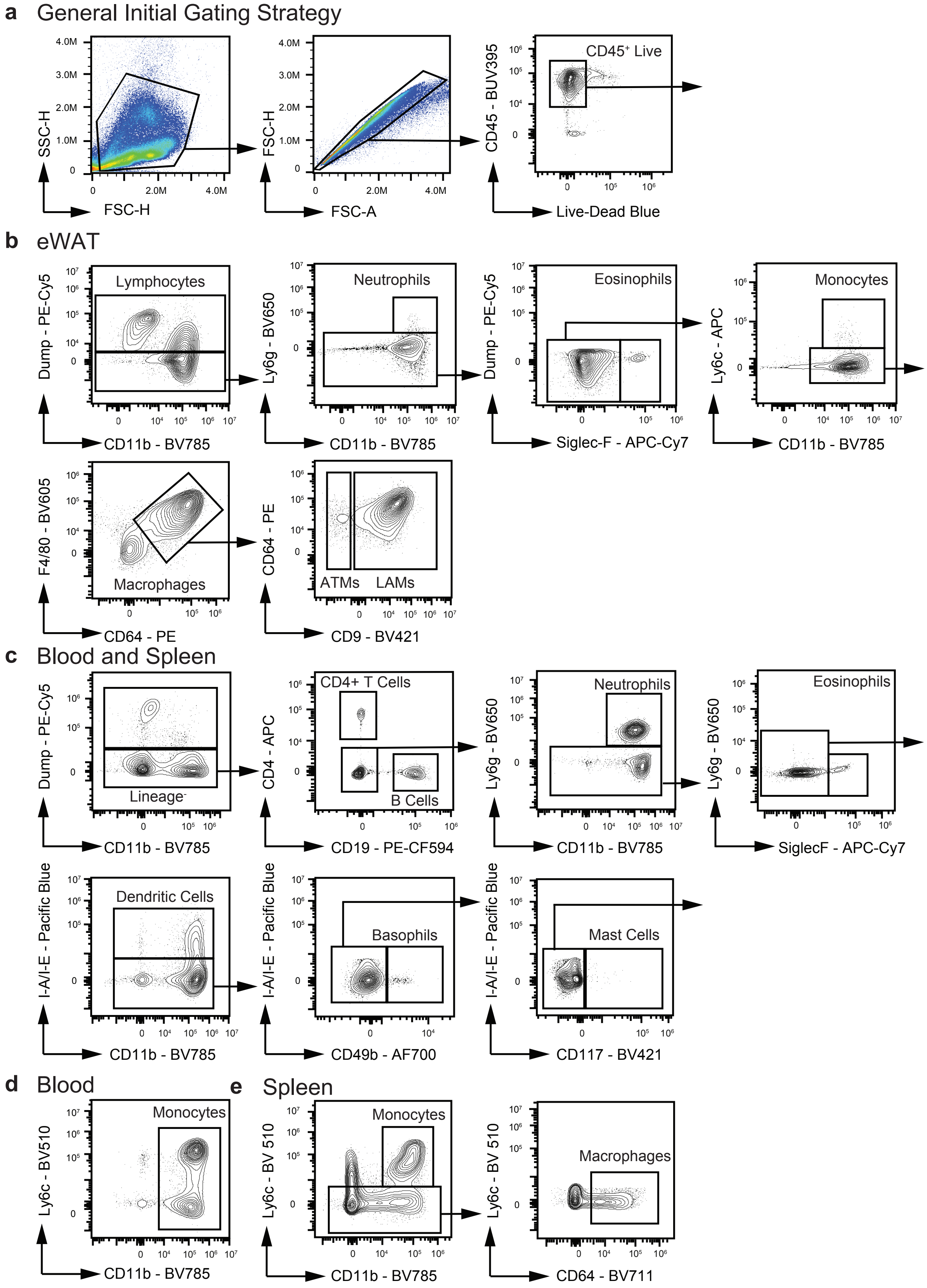

Supplementary Fig. 5: Peripheral flow gating strategies. General initial (a), eWAT (Dump = CD3, B220, and NK1.1) (b), and blood and spleen (Dump = CD8a, NK1.1, Ter119) (c-e) gating strategies.

Paneru & Chini *et al* - Supplementary Figure 6

Bone Marrow Gating Strategy

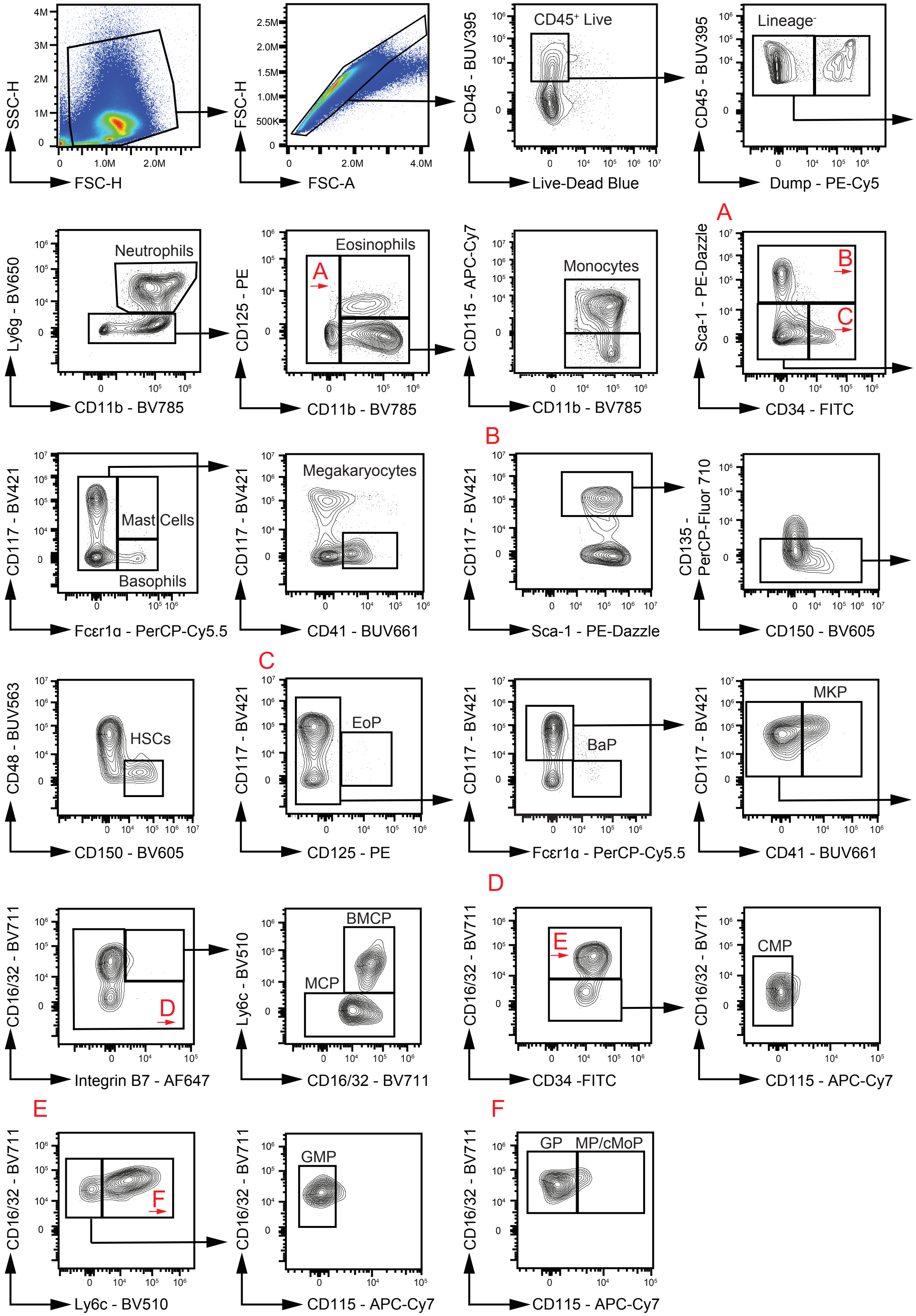

Supplementary Fig. 6: Bone marrow gating strategy. Dump = CD3, NK1.1, Ter119, B220, and IL7R.

Paneru & Chini *et al* - Supplementary Figure 7

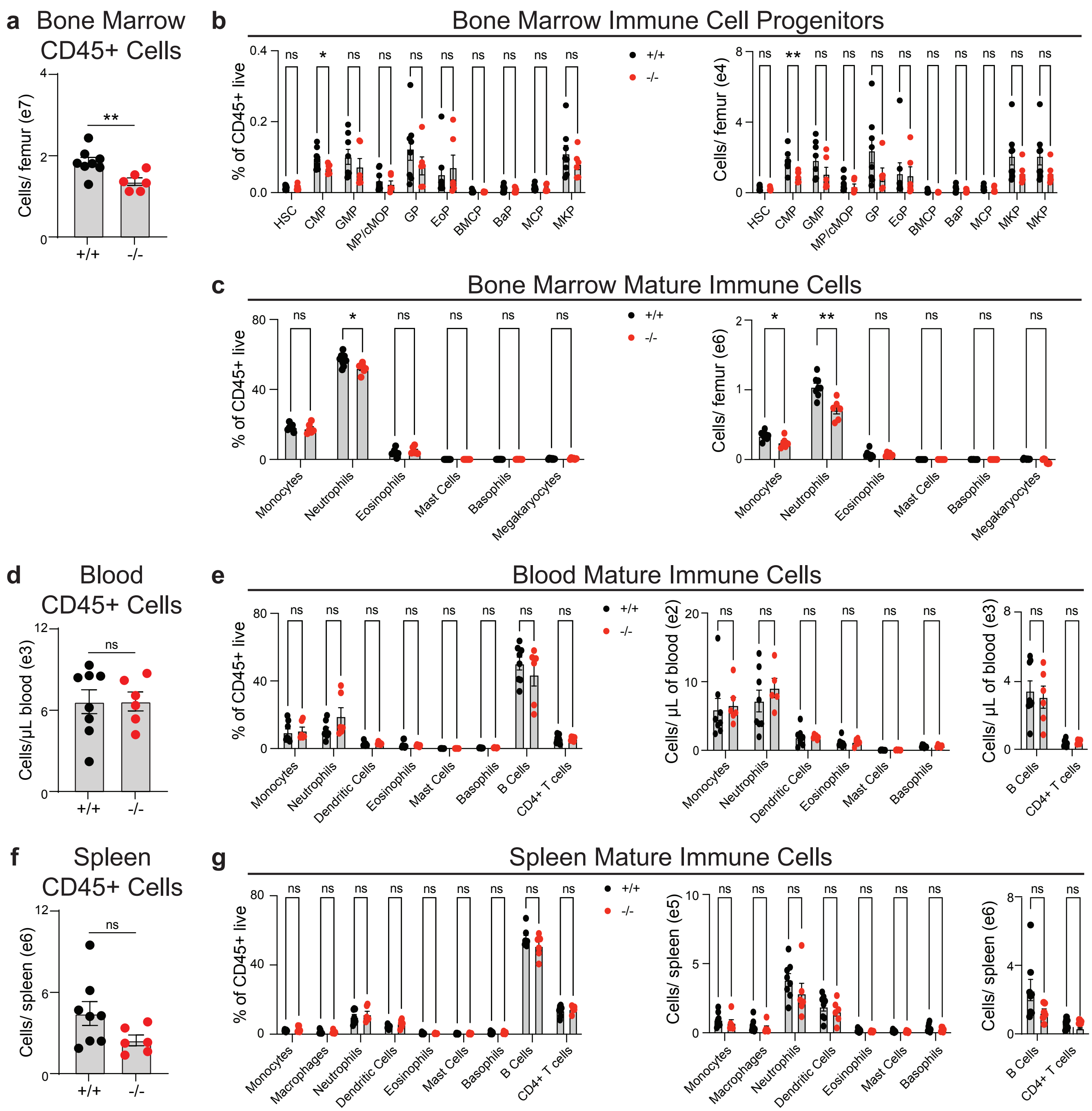

Supplementary Fig. 7: Bone marrow, spleen, and blood immune cell complement of DIO wild-type or miR-6236 deficient mice. a-g, Flow cytometric analysis of immune cells in wild-type littermate (+/+; n = 8) or whole-body miR-6236 knockout (-/-; n = 6) male mice. CD45+ cells (a), immune cell progenitors (b), and mature immune cells (c) in bone marrow (CD45+ Cells, p = 0.0087; CMP frequency, p = 0.0040; CMP counts, p = 0.0084; Neutrophil frequency, p = 0.022; Monocyte counts, p = 0.032; Neutrophil counts, p = 0.0015). d-e, Flow cytometric analysis of CD45+ cells (d) and mature immune cells (e) in blood. f-g, Flow cytometric analysis of CD45+ cells (f) and mature immune cells (g) in spleen. Gating strategies shown in Supplementary Figures 5 & 6. Abbreviations: HSC: hematopoietic stem cell; CMP: Common Myeloid Progenitor; GMP: Granulocyte-Monocyte Progenitor; MP/cMOP: Monocyte Progenitor/Common Monocyte Progenitor; GP: Granulocyte Progenitor; EoP: Eosinophil Progenitor; BMCP: Basophil Mast Cell Progenitor; BaP: Basophil Progenitor; MCP: Mast Cell Progenitor; MKP: Megakaryocyte Progenitor. Data representative of ≥2 experiments. Data presented as mean ± SEM; ns = not significant, $*p < 0.05$, $**p < 0.01$, two-tailed Student's t test.

Paneru & Chini *et al* - Supplementary Figure 8

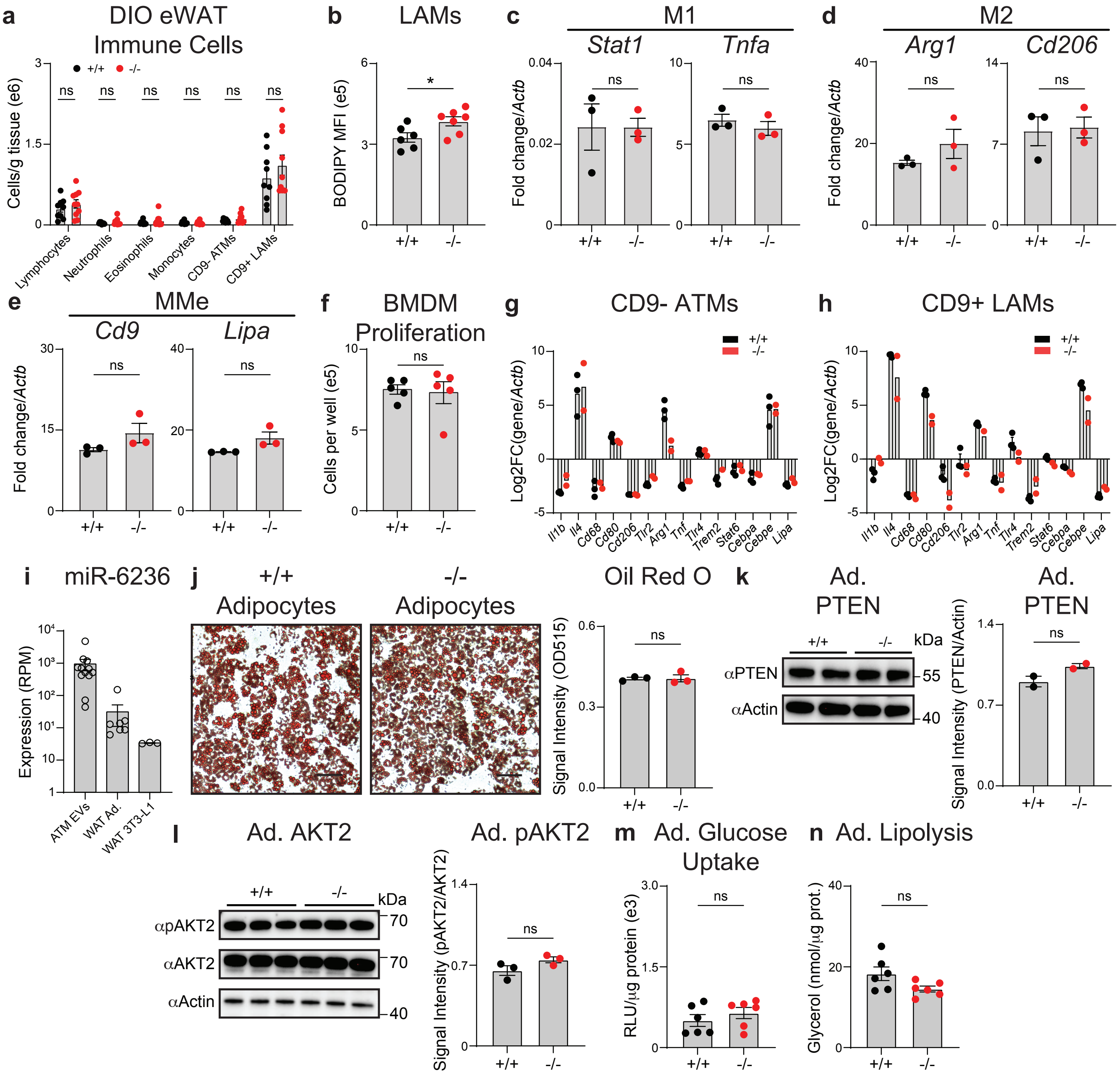

Supplementary Fig. 8: Macrophage and adipocyte-intrinsic effects of miR-6236. a, Number of immune cells determined by flow profiling in eWAT of DIO wild-type (+/+; n = 9) or miR-6236 knockout (-/-; n = 10) male mice. b, Lipid content measured as BODIPY stain MFI (mean fluorescence intensity) in LAMs of DIO male wild-type (+/+; n = 6) or miR-6236 knockout (-/-; n = 7) mice ($p = 0.032$). c-e, Transcript abundance of M1, M2 and metabolic activation (MMe) markers in *ex vivo* differentiated BMDMs from DIO male mice (n = 3 technical replicates/group). f, Cell counts per well of BMDM cultures (n = 5). g-h, Expression of macrophage-related genes in CD9- ATMs (g) and CD9+ LAMs (h) sorted from DIO male wild-type (+/+; n = 3) or miR-6236 knockout (-/-; n = 2) mice. i, Expression level of miR-6236 in ATM-derived EVs (n = 12 biological replicates), white adipose tissue (WAT) adipocytes (Ad.; n = 7 biological replicates), and 3T3-L1 adipocytes (n = 3 technical replicates) as measured by small RNA-seq. j, Oil Red O-stained primary adipocytes differentiated from primary preadipocytes of DIO male mice (left) and quantification of total Oil Red O stain (right) (n = 3, scale bar = 100 μm). k, PTEN Western blot and quantification in *ex vivo* differentiated primary adipocytes (Ad.) from DIO male mice (n = 2). l, AKT2 and pAKT2 Western blot and quantification in *ex vivo* differentiated primary adipocytes from DIO male mice (n = 3). m, Insulin-stimulated glucose (Glu.) uptake in *ex vivo* differentiated primary adipocytes from DIO male mice (n = 6). n, Insulin-stimulated lipolysis suppression in *ex vivo* differentiated primary adipocytes from DIO male mice (n = 6). Data representative of ≥2 experiments. Data presented as mean ± SEM; ns = not significant, $^*p < 0.05$, two-tailed Student's t test.

Paneru & Chini *et al* - Supplementary Figure 9

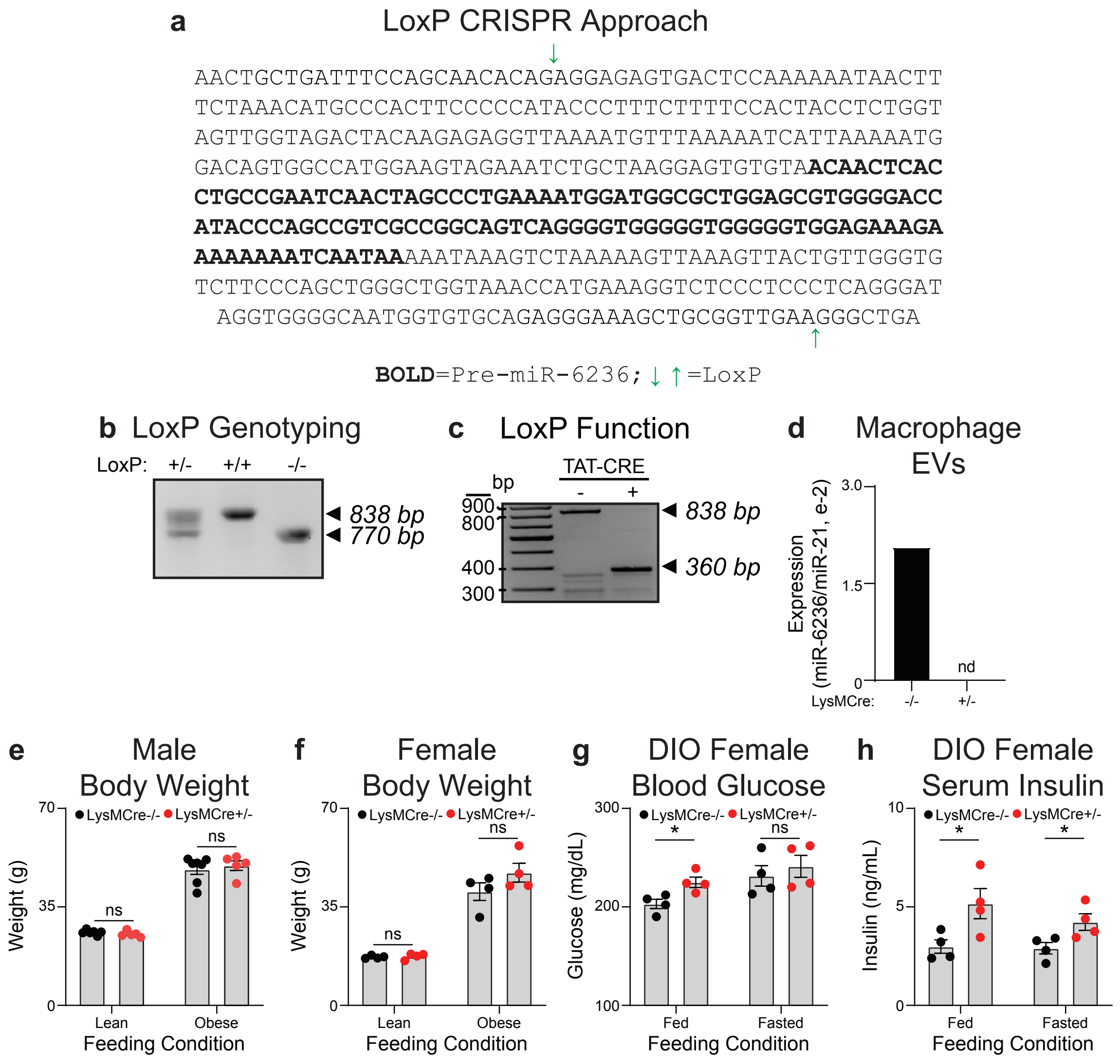

Supplementary Fig. 9: Generation and phenotyping of miR-6236fl/fl mice. a, Genomic sequence of miR-6236 showing pre-miR-6236 sequence (bold) and flanking sequences. Green arrows indicate locations where LoxP sequences were inserted to generate miR-6236fl/fl mice. b, DNA PCR gel image showing the expected band size of the miR-6263 DNA locus in LoxP+/+, LoxP+/-, and LoxP-/- mice. c, DNA PCR gel image showing deletion of miR-6236 DNA locus between inserted LoxP sites in miR-6236fl/fl BMDMs treated with TAT-Cre recombinase *in vitro*. d, miR-6236 expression in EVs of BMDMs derived from miR-6236fl/fl mice. nd = not detected. e, Body weight of miR-6236fl/fl male mice under lean (LysM -/-, n = 6, LysM +/+, n = 5) and DIO (LysM -/-, n = 7, LysM +/+, n = 5) states. f, Body weight of miR-6236fl/fl female mice under lean and DIO states (n = 4 mice/group). g, Fed and fasted (5 hrs.) blood glucose level in DIO miR-6236fl/fl female mice (n = 4 mice /group, Fed, $p = 0.021$). h, Fed and fasted (5 hrs.) serum insulin level in DIO miR-6236fl/fl female mice (n = 4; Fed, $p = 0.040$; Fasting, $p = 0.041$). Data representative of ≥2 experiments. Data presented as mean ± SEM; ns = not significant, $*p < 0.05$, two-tailed Student's t test.

Paneru & Chini *et al* - Supplementary Figure 10

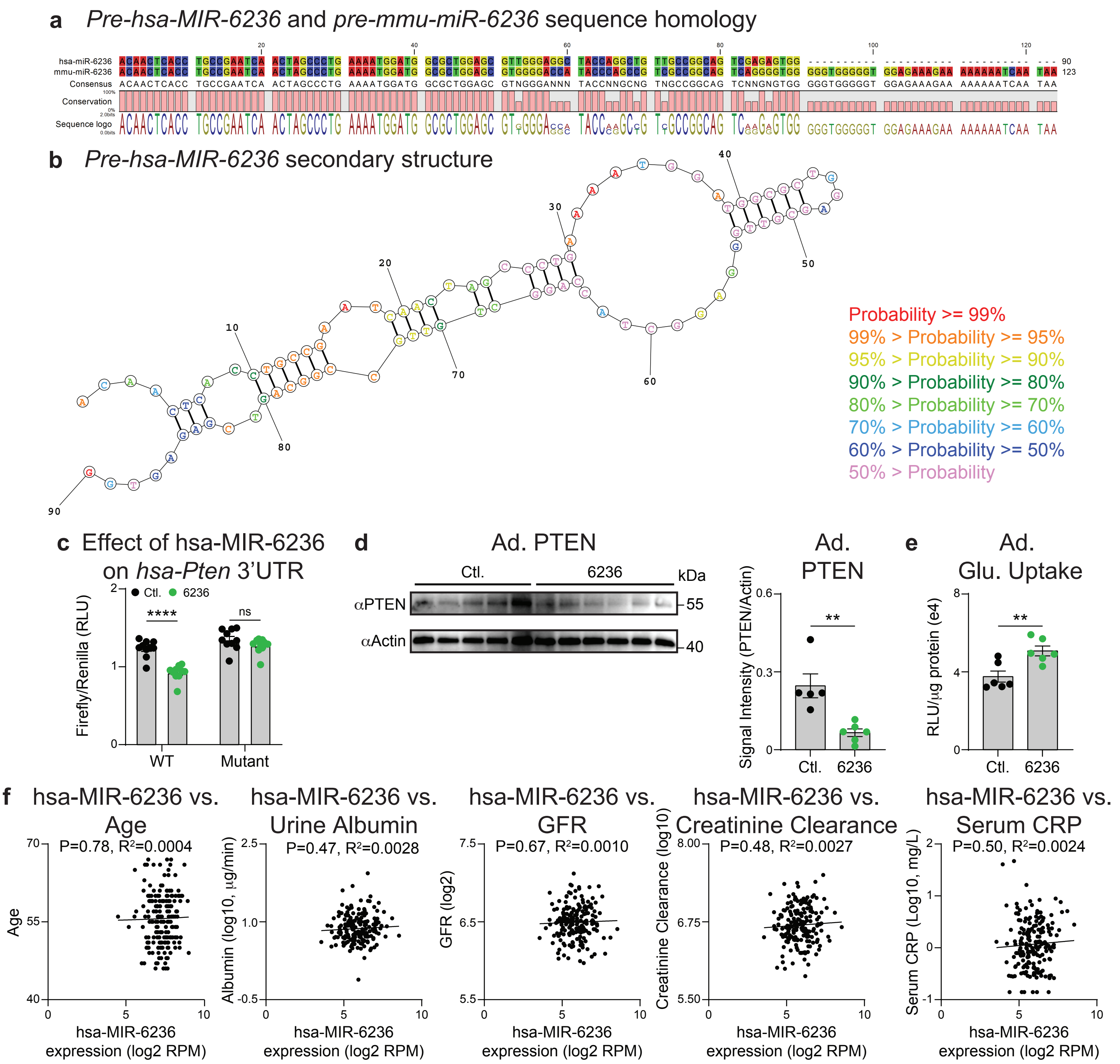

Supplementary Fig. 10: Human and murine miR-6236 sequence homology, pre-hsa-MIR-6236 stem-loop structure, and correlation of hsa-MIR-6236 with clinical and metabolic traits. a, Alignment of human (hsa) and murine (mmu) miR-6236 precursor sequences. b, Stem-loop structure of hsa-MIR-6236 precursor sequence. c, Dual luciferase reporter assay of scrambled control RNA (Ctl.) or hsa-MIR-6236 (6236) binding to a wild-type (WT) hsa-Pten 3'UTR or hsa-Pten 3'UTR with hsa-MIR-6236 binding sites deleted (Mutant) (n = 10; $p < 0.0001$). d, PTEN Western blot and quantification in primary human adipocytes (Ad.) transfected with scrambled control RNA (Ctl.; n = 5) or hsa-MIR-6236 (6236; n = 6) ($p = 0.0029$). e, Insulin-stimulated glucose uptake in primary human adipocytes (n = 6; $p = 0.0061$). f, simple linear regression with two-sided Pearson correlation of hsa-MIR-6236 expression level in subcutaneous adipose tissue with subject age, urine albumin level, glomerular filtration rate (GFR), creatinine clearance rate, and serum C-reactive protein (CRP) (n=189). Panel c-e data representative of ≥2 experiments and presented as mean ± SEM; ns = not significant, **$p < 0.01$, ****$p < 0.0001$, two-tailed Student's t test for panels c-e.
