# Supplementary material for: Myeloid-derived miR-6236 potentiates adipocyte insulin signaling and prevents hyperglycemia during obesity
Source: Nat Commun. 2024 Jun 25;15:5394. doi: 10.1038/s41467-024-49632-z (PMC11199588; doi:10.1038/s41467-024-49632-z)
Supplement: Supplementary file 3 — Description of Additional Supplementary Files [file 41467_2024_49632_MOESM3_ESM.pdf]

Description of Additional Supplementary Files

Supplementary Data 1: Table of key reagents and primers. Table of key reagents, sources, and primers.
