# Supplementary material for: Myeloid-derived miR-6236 potentiates adipocyte insulin signaling and prevents hyperglycemia during obesity
Source: Nat Commun. 2024 Jun 25;15:5394. doi: 10.1038/s41467-024-49632-z (PMC11199588; doi:10.1038/s41467-024-49632-z)
Supplement: Supplementary file 5 — Reporting Summary [file 41467_2024_49632_MOESM5_ESM.pdf]

nature portfolio

Corresponding author(s): David A. Hill, MD, PhD

Last updated by author(s): May 26, 2024

Reporting Summary

Nature Portfolio wishes to improve the reproducibility of the work that we publish. This form provides structure for consistency and transparency in reporting. For further information on Nature Portfolio policies, see our Editorial Policies and the Editorial Policy Checklist.

Statistics

For all statistical analyses, confirm that the following items are present in the figure legend, table legend, main text, or Methods section.

n/a	Confirmed	
☐	☒	The exact sample size (n) for each experimental group/condition, given as a discrete number and unit of measurement
☐	☒	A statement on whether measurements were taken from distinct samples or whether the same sample was measured repeatedly
☐	☒	The statistical test(s) used AND whether they are one- or two-sided<br>*Only common tests should be described solely by name; describe more complex techniques in the Methods section.*
☒	☐	A description of all covariates tested
☒	☐	A description of any assumptions or corrections, such as tests of normality and adjustment for multiple comparisons
☐	☒	A full description of the statistical parameters including central tendency (e.g. means) or other basic estimates (e.g. regression coefficient) AND variation (e.g. standard deviation) or associated estimates of uncertainty (e.g. confidence intervals)
☐	☒	For null hypothesis testing, the test statistic (e.g. F, t, r) with confidence intervals, effect sizes, degrees of freedom and P value noted<br>*Give P values as exact values whenever suitable.*
☒	☐	For Bayesian analysis, information on the choice of priors and Markov chain Monte Carlo settings
☒	☐	For hierarchical and complex designs, identification of the appropriate level for tests and full reporting of outcomes
☐	☒	Estimates of effect sizes (e.g. Cohen's d, Pearson's r), indicating how they were calculated

Our web collection on statistics for biologists contains articles on many of the points above.

Software and code

Policy information about availability of computer code

Data collection	No software was used.
Data analysis	Analysis was performed in CLC Genomics or GraphPad using standard software.

For manuscripts utilizing custom algorithms or software that are central to the research but not yet described in published literature, software must be made available to editors and reviewers. We strongly encourage code deposition in a community repository (e.g. GitHub). See the Nature Portfolio guidelines for submitting code & software for further information.

Data

Policy information about availability of data

All manuscripts must include a data availability statement. This statement should provide the following information, where applicable:

- Accession codes, unique identifiers, or web links for publicly available datasets
- A description of any restrictions on data availability
- For clinical datasets or third party data, please ensure that the statement adheres to our policy

In-lab generated raw small RNA sequencing data from CD9+ ATM EVs fractions are deposited in Mendeley under accession number doi: 10.17632/cbszxmd63x.1 (https://data.mendeley.com/datasets/cbszxmd63x/1). NCBI locations of raw public data used in this study are as follows: murine tissue and ATM derived EV small RNA-seq [(https://www.ncbi.nlm.nih.gov/geo/query/acc.cgi?acc=GSE119661, GSE119661), (https://www.ncbi.nlm.nih.gov/geo/query/acc.cgi?acc=GSE142677, GSE142677), (https://www.ncbi.nlm.nih.gov/geo/query/acc.cgi?acc=GSE97652, GSE97652)]; murine adipose tissue argonaute HITS-CLIP (https:// www.ncbi.nlm.nih.gov/geo/query/acc.cgi?acc=GSE142677, GSE142677); murine Kupffer cell small RNA-seq (https://www.ncbi.nlm.nih.gov/geo/query/acc.cgi?

acc=GSE160016, GSE160016); young (6 month old) and aged (24 month old) murine serum small RNA-Seq (https://www.ncbi.nlm.nih.gov/geo/query/acc.cgi?acc=GSE76442, GSE76442); murine peritoneal macrophage PPAR ChIP-seq (https://www.ncbi.nlm.nih.gov/geo/query/acc.cgi?acc=GSM3022232, GSM3022232); murine LAM ATAC-seq (https://www.ncbi.nlm.nih.gov/geo/query/acc.cgi?acc=GSE113583, GSE113583); 3T3-L1 and primary adipocyte small RNA-Seq [(https://www.ncbi.nlm.nih.gov/bioproject/PRJEB20090/, PRJEB20090) (https://www.ncbi.nlm.nih.gov/bioproject/PRJEB25978/, PRJEB25978)]; Human Drosha KO RNA-seq (https://www.ncbi.nlm.nih.gov/bioproject/PRJNA282167/, PRJNA282167); Human serum small RNA-seq (https://www.ncbi.nlm.nih.gov/bioproject/PRJEB21747/, PRJEB21747); Human adipose tissue small RNA-seq (https://www.ncbi.nlm.nih.gov/geo/query/acc.cgi?acc=GSE45159, GSE45159); Human argonaute HITS-CLIP [(https://www.ncbi.nlm.nih.gov/geo/query/acc.cgi?acc=GSE41272, GSE412720 (https://www.ncbi.nlm.nih.gov/geo/query/acc.cgi?acc=GSE41357, GSE41357) and human adipocytes ATAC-seq (https://www.ncbi.nlm.nih.gov/geo/query/acc.cgi?acc=GSE178796, GSE178796)

Research involving human participants, their data, or biological material

Policy information about studies with human participants or human data. See also policy information about sex, gender (identity/presentation), and sexual orientation and race, ethnicity and racism.

Reporting on sex and gender	N/A
Reporting on race, ethnicity, or other socially relevant groupings	N/A
Population characteristics	N/A
Recruitment	N/A
Ethics oversight	N/A

Note that full information on the approval of the study protocol must also be provided in the manuscript.

Field-specific reporting

Please select the one below that is the best fit for your research. If you are not sure, read the appropriate sections before making your selection.

- [x] Life sciences
- [ ] Behavioural & social sciences
- [ ] Ecological, evolutionary & environmental sciences

For a reference copy of the document with all sections, see nature.com/documents/nr-reporting-summary-flat.pdf

Life sciences study design

All studies must disclose on these points even when the disclosure is negative.

Sample size	We did not pre-calculate sample size.
Data exclusions	None.
Replication	Experiments were replicated as detailed in the figure legends.
Randomization	In experiments using wild-type mice, mice were randomized into groups.
Blinding	None.

Reporting for specific materials, systems and methods

We require information from authors about some types of materials, experimental systems and methods used in many studies. Here, indicate whether each material, system or method listed is relevant to your study. If you are not sure if a list item applies to your research, read the appropriate section before selecting a response.

Materials & experimental systems

n/a	Involved in the study	
[ ]	[x]	Antibodies
[ ]	[x]	Eukaryotic cell lines
[x]	[ ]	Palaeontology and archaeology
[ ]	[x]	Animals and other organisms
[ ]	[x]	Clinical data
[x]	[ ]	Dual use research of concern
[x]	[ ]	Plants

Methods

n/a	Involved in the study	
[x]	[ ]	ChIP-seq
[ ]	[x]	Flow cytometry
[x]	[ ]	MRI-based neuroimaging

Antibodies

Antibodies used	Anti-Phospho Akt2 (Ser474) (D3H2) Cell Signaling 8599S<br>Anti-Akt2 (D6G4) Cell Signaling 3063S<br>Anti-Pten (A2B1) Santa Cruz Biotech. sc-7974<br>Anti-actin Sigma Aldrich A2066-100UL<br>Anti-mouse IgG HRP Cell Signaling 7076S<br>Anti-rabbit IgG HRP Novus Biologicals HAF008<br>Cd45 Rat anti Mouse, BUV395, Clone: 30 F11, BD BD-bioscience 564279<br>BV421 Rat Anti-Mouse CD9 Clone KMC8 BD-bioscience 564235Â<br>Anti-CD86 Rat Monoclonal Antibody Biolegend 105039<br>Brilliant Violet 605, anti-mouse F4/80 Antibody Biolegend 123133<br>Brilliant Violet 650, anti-mouse Ly-6G BioLegend 127641<br>Anti-CD11b Rat Monoclonal Antibody (Brilliant Violet-785) BioLegend 101243<br>BODIPY Thermo Scientific D3922<br>PerCP/Cyanine5.5 anti-mouse CD68 Antibody Biolegend 137009<br>PE anti-mouse CD64 (FceRI) Antibody Biolegend 164403<br>Pe/dazzle- 594 anti-mouse I-A/I-E Biolegend 107647<br>Cd11c Monoclonal Antibody (N418), PE-Cyanine5.5 Thermo Scientific 35-0114-82<br>PE/Cyanine7 anti-mouse CD206 Biolegend 141720<br>Anti-Ly-6C Rat Monoclonal Antibody (APC (Allophycocyanin)) Biolegend 128015<br>Cd197 (CCR7) Monoclonal Antibody (4B12), Alexa Fluor 700 Thermo Scientific 56-1971-82<br>Siglec F Rat anti Mouse, APC Cy7, Clone: E50 2440 Thermo Scientific BDB565527<br>Phospho-HSL (Ser660) antibody Cell Signaling 4126<br>HSL antibody Cell Signaling 4107<br>CD9 Antibody BD Biosciences 553758
Validation	These antibodies are commonly used clones purchased from reputable companies and were utilized following recommended protocols.

Eukaryotic cell lines

Policy information about cell lines and Sex and Gender in Research

Cell line source(s)	3T3-L1 ATCC CL-173<br>293T ATCC CRL-3216
Authentication	Cell lines were purchased directly from ATCC.
Mycoplasma contamination	Cell lines were not tested for mycoplasma.
Commonly misidentified lines<br>(See ICLAC register)	N/A

Animals and other research organisms

Policy information about studies involving animals; ARRIVE guidelines recommended for reporting animal research, and Sex and Gender in Research

Laboratory animals	C57BL/6J littermate WT controls<br>Mouse/B6: miR-6236 KO (whole body)<br>Mouse/B6: miR-6236 loxP/loxP<br>LysM Cre: B6.129P2-Lyz2tm1(cre)Ifo/J Jackson Laboratory Stock no. 004781
Wild animals	N/A
Reporting on sex	Effects of biologic sex were considered and sex-disaggregated data is presented in the manuscript.
Field-collected samples	N/A
Ethics oversight	CHOP IACUC approved these studies.

Note that full information on the approval of the study protocol must also be provided in the manuscript.

Clinical data

Policy information about clinical studies

All manuscripts should comply with the ICMJEguidelines for publication of clinical research and a completedCONSORT checklist must be included with all submissions.

Clinical trial registration	N/A
Study protocol	N/A
Data collection	N/A
Outcomes	N/A

Plants

Seed stocks	N/A
Novel plant genotypes	N/A
Authentication	N/A

Flow Cytometry

Plots

Confirm that:

- [x] The axis labels state the marker and fluorochrome used (e.g. CD4-FITC).
- [x] The axis scales are clearly visible. Include numbers along axes only for bottom left plot of group (a 'group' is an analysis of identical markers).
- [x] All plots are contour plots with outliers or pseudocolor plots.
- [x] A numerical value for number of cells or percentage (with statistics) is provided.

Methodology

Sample preparation	eWAT was dissected, weighed, and digested with collagenase and DNase to isolate the SVF fraction
Instrument	SVF fraction was stained with fluorochrome-conjugated monoclonal antibodies, quantified using AURORA flow cytometer
Software	Data was analyzed in FlowJo
Cell population abundance	N/A
Gating strategy	Cells were gated on live, CD45+.

- [x] Tick this box to confirm that a figure exemplifying the gating strategy is provided in the Supplementary Information.
